# SiaN-VO: Siamese Network for Visual Odometry †

**DOI:** 10.3390/s24030973

**Published:** 2024-02-02

**Authors:** Bruno S. Faiçal, Cesar A. C. Marcondes, Filipe A. N. Verri

**Affiliations:** Computer Science Division, Aeronautics Institute of Technology—ITA, Sao Jose dos Campos 12228-900, SP, Brazil; cmarcondes@ita.br

**Keywords:** visual odometry, drone, autonomous flight

## Abstract

Despite the significant advancements in drone sensory device reliability, data integrity from these devices remains critical in securing successful flight plans. A notable issue is the vulnerability of GNSS to jamming attacks or signal loss from satellites, potentially leading to incomplete drone flight plans. To address this, we introduce SiaN-VO, a Siamese neural network designed for visual odometry prediction in such challenging scenarios. Our preliminary studies have shown promising results, particularly for flights under static conditions (constant speed and altitude); while these findings are encouraging, they do not fully represent the complexities of real-world flight conditions. Therefore, in this paper, we have furthered our research to enhance SiaN-VO, improving data integration from multiple sensors and enabling more accurate displacement predictions in dynamic flight conditions, thereby marking a significant step forward in drone navigation technology.

## 1. Introduction

Unmanned Aerial Vehicles (UAVs) use Global Navigation Satellite System (GNSS) signals as their primary location tool. Knowing the global position (latitude and longitude coordinates) allows the flight control system to perform missions in outdoor environments. However, this system is susceptible to various types of attacks and interruptions in signal reception [1,2].

During the aircraft’s flights, particularly during search and rescue (SAR) missions, an accurate position estimation of the aircraft is crucial, even in the absence of Global Navigation Satellite System (GNSS) data [3]. Research in this domain has explored various methodologies, including sensor fusion, the use of Inertial Measurement Units (IMUs), and image-based inference techniques. Notably, image-based navigation employs multiple strategies for location estimation, such as recognizing environmental landmarks and analyzing optical flow [4,5,6,7]. However, these image-based methods typically demand extensive computational resources due to their reliance on intricate image processing pipelines.

In the existing literature, various studies have proposed image-based methods for estimating displacement from a reference point, such as the initial position, to facilitate collision avoidance [8,9,10]. However, there needs to be more research regarding the exclusive use of Convolutional Neural Networks (CNNs) and dataflow for global position estimation of Unmanned Aerial Vehicles (UAVs). Hence, using lightweight Artificial Neural Network (ANN) models in image-based approaches could reduce computational costs during the inference phase.

Moreover, CNNs have become a cornerstone in computer vision, often serving as a fundamental component in image processing within machine learning frameworks [11,12]. Compared to traditional fully connected ANN layers, CNNs are more efficient in processing local spatial patterns in images and require significantly fewer parameters [13].

Building on this, we introduce the Siamese Neural Network for Visual Odometry (SiaN-VO), a method capable of determining displacement between a pair of sequential images captured by a drone. SiaN-VO represents an advanced version of a method previously discussed in another study [14]. It uses inferred displacement and the haversine formula to calculate the UAV’s new geographic coordinates (latitude and longitude). We assume that flight direction and vehicle altitude are acquired from other sensors, such as a compass and a radio altimeter. This novel approach enables displacement inference during flights with varying altitudes, addressing the limitations of earlier methods that required constant altitude flights. We also keep the number of parameters in the network low, so that we can enable the future implementation of the solution in an embedded system on the aircraft.

The remainder of this paper is organized as follows: In Section 2, we review the relevant scientific literature that relates to our proposal. Section 3 provides a comprehensive description of the system we propose. Section 4 elaborates on the methodology employed in our experiments, including details about the dataset, the training phase of our proposed method, and the testing procedures. Section 5 presents the results we obtained. The paper concludes with Section 6, wherein we discuss our conclusions and directions for future research.

## 2. Scientific Literature

Numerous works in the literature have highlighted the importance of investigating methods and approaches for different vehicles (ground or air) to have location information without the dependency on external sources [8,9,10]. In general, these works investigate approaches that allow vehicles to move safely and reach their goal.

Visual approaches, such as visual odometry, have attracted attention for several reasons, among them, price, accessibility, accuracy, and the independence of external signals, as in the case of GNSS-based methods [15,16]. Visual odometry is the process of estimating the motion of an agent (e.g., vehicle, human, and robot) using only the input from a single or multiple cameras [17].

There are works describing good results in merging visual odometry information with other information to achieve better accuracy in location inference [18,19]. The results show that merging information from visual odometry with other sensors can increase the accuracy of the positioning and movement information. Visual odometry commonly uses an important concept called optical flow.

Optical flow has been used to detect the motion of objects and scenery to help autonomously drive vehicles and avoid collisions [20]. An example of this scenario can be seen in [21], where the proposed method (named LiteFlowNet2) is evaluated on datasets from different contexts. The MPI Sintel dataset is a dataset derived from the open-source 3D animation short film Sintel. In this setting, the method receives a pair of sequential images, and its output is a segmented image of the regions occupied by the characters’ movements in the time interval between the images received as input. Another dataset used is KITTI [20,22], which is a set of images captured by a car on urban routes. In this dataset, the method is evaluated for the goal of detecting the surrounding scenery in motion.

Works that use CNN to estimate the position and displacement of unmanned aerial vehicles are found in the scientific literature [14,23,24]. Olawoye and Gross [23] propose applying a deep learning approach to 3D object detection to calculate the relative position of an Unmanned Aerial Vehicle (UAV). However, this approach requires an Unmanned Ground Vehicle (UGV) equipped with a LiDAR sensor in order to operate in GPS-denied environments. In a study by Araveti et al. [24], another method is proposed to estimate drone displacement, but this method is not designed to deal with altitude variation. This weakness is also observed in the paper originally published by the authors of the current work [14], where the proposed method was unable to deal with altitude variation. In view of this, it is possible to note that the current work proposes a method capable of overcoming the obstacles of altitude variation and the requirement for additional vehicles.

One can observe in the literature works that use the concepts above to estimate the movement of unmanned aerial vehicles [25,26]. The proposed methods can be used in outdoor and indoor environments.

However, it is common for navigation systems to use geographic coordinate information to manage UAV flights. Considering this context, we were not able to find works with the objective of inferring the geographic coordinates of UAVs during flight.

## 3. Siamese Network for Visual Odometry (SiaN-VO)

In this section, we describe the proposed new ANN model and its training procedure. It is important to emphasize that the proposed method presents the evolution of previously published work and the overcoming of the limitation on flight dynamics.

### Network Architecture

**Figure 1:**
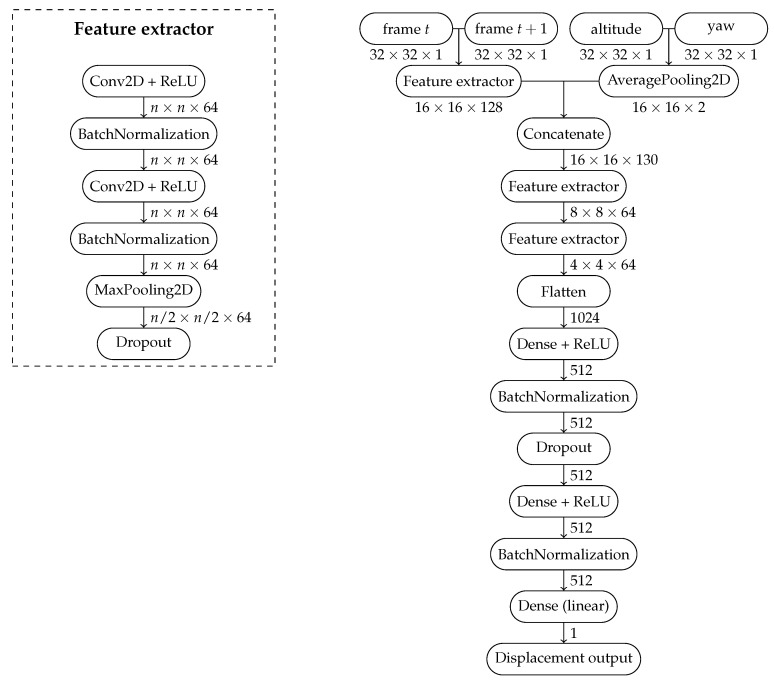
Architecture of the Siamese Network for Visual Odometry (SiaN-VO).

The proposed neural network model must be able to receive two images and two other matrices as input. One of them must contain the altitude value, while the other matrix must contain data from a sensor that captures the aircraft yaw variable (the angle at which the front of the drone is facing, based on magnetic north.). The matrices have the same dimensions as the images and their values are repeated in all the cells. The transformation of the unit values into matrices with the value repeated in all the cells aims to provide stimulation similar to that of the images. This prevents the altitude and yaw data stimuli from being ignored because they are weak stimuli compared to the number of values in the image pair. The detailed architecture can be seen in Figure 1. The total number of trainable params is 2,004,801, i.e., around 7.65 MB of data.

Pixel values are normalized between 0 and 1 for the image pairs. Yaw is also normalized between 0 and 1, where 0.5 denotes magnetic north, 0 denotes −180 degrees, and 1 denotes 180 degrees. Altitude input that the aircraft flies with maximum altitude *h* from the ground. Thus, we divide the input by *h* so its maximum value is also 1.

Our network can be divided into two parts:

1. In the first part, we have a Siamese network, where the model receives a pair of images taken at consecutive time steps, and these images are passed through identical neural network layers and weights. We chose such a design assuming that the images could be processed independently. The neural network can find useful coarse patterns in them, like edges, before joining both images. Parallel to the Siamese network, there is an AveragePooling step that will adjust the dimensions of the altitude and yaw matrices to the same dimensions as the feature maps resulting from the Siamese network.
2. The results of the Siamese network and AveragePooling are concatenated, effectively overlaying the image maps and the two complementary data matrices. The concatenated feature maps then pass through a normal CNN pipeline, ending in a prediction head containing three fully connected layers, the last of which has only a single output—the displacement output.

During the search for the best hyperparameters for the neural network, we find that larger filter sizes work best for our problem, and we ultimately employ 7×7 filters throughout the network. Apart from the filter size, our design choices took inspiration from the VGGNet architecture [27]: at every new set of convolutional layers, we double the number of filters, as well as reduce, by half, the spatial dimensions of the image maps by means of applying MaxPooling layers with a kernel size of 2×2. In summary, our model is composed of six convolutional layers (two layers in the Siamese network and four sequential convolutional layers) and three fully connected layers. Dropout layers are set at 20%. In total, the neural network is nine layers deep. It is important to note that the Siamese network is encapsulated in the figure within the “Feature extractor” element.

Once the model is to be employed in a small unmanned aircraft, computational power and storage are important restrictions. With this in mind, we designed our neural network to receive gray-scale images of dimensions 32×32. The use of gray-scale images should suffice for the task of displacement prediction, as the color information transmitted by the red, green, and blue channels should not give any significant insights into the movement of the vehicle. We highlight that we have reduced the size of the images used compared to the method proposed previously, allowing for a less computationally expensive execution.

## 4. Methodology for the Experiments

In this section, we will describe the dataset used and the methodology employed in the training and testing stages to evaluate SiaN-VO.

### 4.1. Dataset

In order to develop a large-scale simulated dataset for the task of UAV displacement estimation, we leverage the capabilities of AirSim [28], which is an open-source simulator for autonomous vehicles, including self-driving cars and drones.

**Figure 2:**
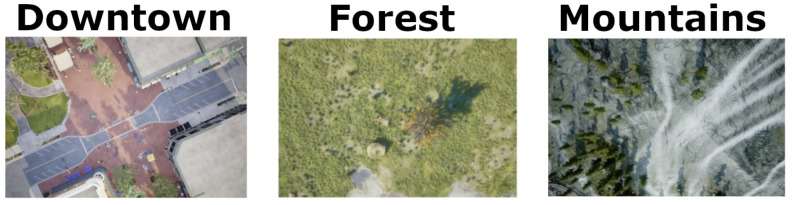
Examples of images considering the environments in which the flights were simulated.

We simulated drone flying in three different scenarios: a mountainous arctic region (mountains), a tropical forest (forest), and a city with a green area (downtown). These scenarios include artifacts like lakes and rivers, as well as different sizes of streets, buildings, and vegetation. Besides the inherent heterogeneity of these choices of maps, we also varied the weather conditions, adding dust, mist, and rain, creating a diversified range of settings. This variability in scenarios is important if we want the machine learning model trained on this data to be able to generalize well to scenes never seen before, which is paramount once we employ these models in real-world applications. We also vary the flight dynamics and characteristics such as altitude, acceleration, direction, and environment. Figure 2 shows an example of the images in the dataset.

Table 1 shows details of the flights simulated in AirSim. The length of the flights varies, as do their maximum and minimum altitudes. It is important to note that the “map” column refers to the region in which the flight took place. Thus, the environment may be forest, but in regions other than forest. It is important to note that, in some cases, the number of flights is less than the standard 50 flights. This is because the map we used was smaller, and we did not want to use it to exhaustion.

The images taken from the drone have a resolution of 720×480 pixels. Even though most applications may need a smaller resolution, the operation of resizing in the data pipeline is very cheap, and on the plus side, the choice of a large resolution gives the user a choice to apply operations of data augmentation, like random-cropping, which need an image larger than the input of the network. The use of data augmentation operations in the preprocessing step can greatly increase the generalization ability of the models trained, as well as virtually increasing the dataset size.

The images are taken from a camera mounted under the vehicle, pointing vertically to the ground. In this configuration, the captured images should obtain the maximum information about the drone movement in the horizontal plane. Additionally, the images are taken with approximately 3 frames per second, which is a slow rate but sufficient to obtain enough superposition between each pair of images, as well as easy to replicate.

Unlike the previous work, the current study shows variation in the drone’s horizontal position and also in its altitude. In addition, the speed sampled for each flight segment comes from a uniform distribution.

Furthermore, each image has an associated file with the ground truth information of the complete status of the drone at the instant the picture was taken, which includes linear and angular speeds, linear and angular accelerations, position, latitude and longitude, and attitude of the vehicle. We explicitly annotated the images with this set of information for the prediction of the vehicle displacement.

### 4.2. Training

For training our model, we employed ReLU activation functions throughout the network, except in the last layer, where we used a linear activation function. Additionally, we used batch normalization layers after every weight layer and dropout layers with a probability of 0.2.

We trained the network on the Tensorflow framework and applied the Adam optimizer with a learning rate of 0.001. For our loss function, we used mean squared error, and we trained the network for 30 epochs. At the end of each epoch during the training stage, the model was evaluated against a validation group. The model that showed an improvement in the value of the metric (considering the validation group) was saved as the best model found. This was the model used for the testing stage.

The dataset was divided into three sets for training: training, validation, and testing. To make up the test set, one flight made on each map of each environment was preserved with the complete sequence of images. In this way, the prediction and its impact during the flight could be evaluated. For the other sets, 80% was set aside for the training set and 20% for the validation set. In the training stage, the sets used were the training and validation sets. Thus, the training set was presented to the model, and at the end of each epoch, the model was evaluated on the validation set. If it showed an improvement in the value of the metric, the model was saved as the best model found so far.

Additionally, during training, the image pairs were shuffled before being presented to the neural network: This ensures that we do not feed correlated data to the model, as the image pairs seen in a single batch will not all be from the same flight or will not have been taken in a sequence by the camera mounted on the drone.

### 4.3. Test

The model generated in the training stage was evaluated in the test stage. In this stage, we used one route from each map (and from each environment) to predict displacement, considering execution during a flight. Table 2 shows the size of each route used in this stage. The name of the route was composed of the name of the environment and the conversion of the map into a numerical value (i.e., A is 1 and B is 2).

Three levels of prediction were made for the final parts of each route. This approach allowed us to better represent the negative impact of prediction on the completion of the mission. The three levels of prediction corresponded to 20%, 40%, and 60%. Finally, the flight was also predicted for the entire route, assuming that only the initial displacement coordinate was known.

It is important to note that although the drone is subject to unknown influences (such as weather conditions) during flight, causing it to move in an undesired direction, we used the yaw (these data can be provided by sensors such as a gyroscope) data to calculate the drone’s new geographical position. This approach allowed us to use the geographical position reported by the drone’s flight system as the “expected position”. The geographical position indicated in our proposal is the “inferred position”, which is used by the drone if there is no GNSS signal. By using data from different sources, we aimed to maintain the integrity of the evaluation stage.

## 5. Results

We emphasize that we conducted experiments to assess the proposed method’s ability to estimate the displacement based on the received inputs. Therefore, the method was used to infer the aircraft’s displacement, and we calculated the new geographical coordinates (lat and long). In this case study, we considered that heading and altitude are obtained by other variables and sensors, such as yaw and radio altimeter.

**Figure 3:**
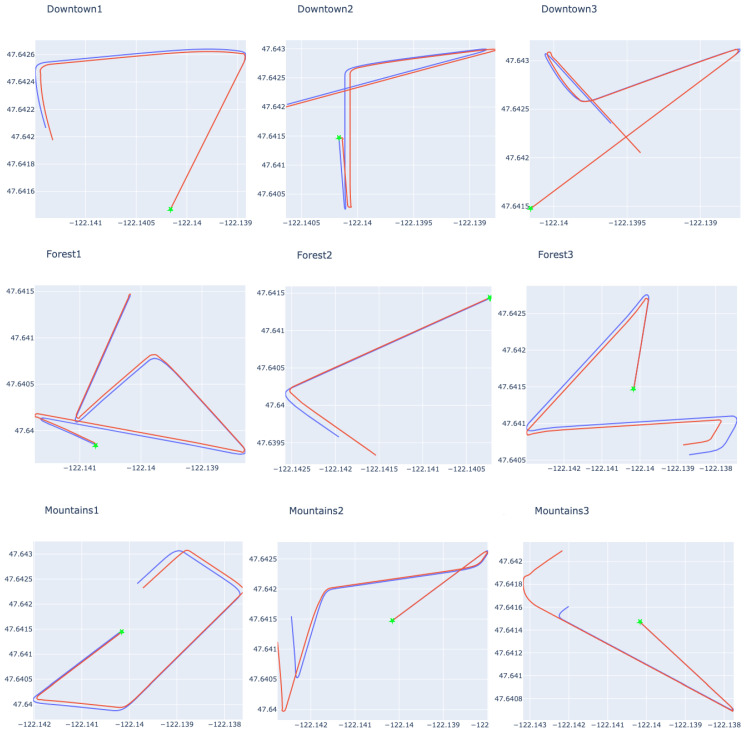
Example of expected routes (blue line) compared with routes that used prediction (red line) for 20% of the flight. The green dot signals the starting position of the flight.

Firstly, Figure 3 shows the routes used in this test stage. The green dot on each route indicates the starting point of the flight. The blue line outlines the expected route, while the red line represents the route taken using the displacement estimation model. In the flights shown in this picture, doom was suffered in the final 20% of the flight. It is important to note that the flights used speed variations, meaning that the distance covered during the prediction varied in size.

More details of the flights can be seen in Table 3, which summarizes the size of the route, the number of predictions made, the distance of the drone’s final position from the expected position, and the average distance between each inferred and expected geographical positions of the drone. It should be noted that this last piece of information is calculated based only on the period in which the displacement was inferred. Table 4, Table 5 and Table 6 present similar information to Table 3. This makes it possible to compare the changes that occurred at different points in the prediction.

**Figure 4:**
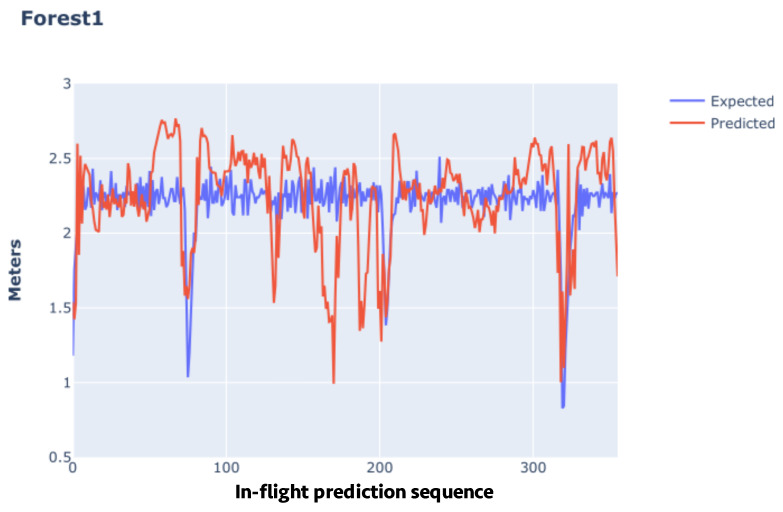
Predicted and expected displacement values along the route Forest1.

In these results, it was impossible to find a clear link between the number of predictions and the metrics relating to comparing the final position and the average positional error during in-flight predictions. In other words, even if the number of predictions is higher on one flight than on another, it does not mean the errors will also be higher. This growth characteristic between the metrics mentioned could not be affirmed when we analyzed the increase in the prediction period considering isolated flights. This behavior occurs because, presumedly, the errors are generated by a normal distribution with the center close to the expected value, causing predictions with positive (greater than expected) and negative (less than expected) error polarities. Figure 4 makes it possible to compare the predicted values with the expected values, preserving the order in which the predictions were made during the flight.

With the variation in environments and routes used in the experiments presented, the SiaN-VO method is capable of predicting displacement in a variety of conditions (even with a change in altitude). Another interesting feature presented by the method in the data set is its ability not to accumulate noise in its prediction.

## 6. Conclusions

Inferring the position of a UAV with high accuracy without the use of a GNSS is an obstacle to several studies in the scientific literature. The increasing evolution of UAVs and the high range of possible contexts in which they can be applied further highlights the need for independence from the GNSS signals for safe navigation.

The evolution provided by the proposed new architecture, giving rise to the SiaN-VO method, could infer the drone’s displacement and allow the value to be used to calculate the new geographical position. SiaN-VO predicts based on a sequential pair of ground images, altitude and yaw data.

The results suggest that noise does not accumulate (or is imperceptible) in the predictions during flights on the routes described. Although we bear in mind that it is possible that noise accumulation becomes noticeable on long journeys and that this characteristic needs to be investigated further, we would like to emphasize that this characteristic is exciting and makes us believe that the SiaN-VO method is a promising approach in the field of visual odometry.

Furthermore, it is important to emphasize that the SiaN-VO method overcame the obstacle of predicting drone displacement on routes with altitude variation. The successful displacement prediction in these flight characteristics means that SiaN-VO surpasses the previous proposal, which exclusively uses images. Another point that surpasses the current approach is the possibility of using images with smaller dimensions (1/4 of the previous size), which allows for less computational processing.

Given the results obtained in this work, we aim to advance our studies in the following directions:

- Evaluate the performance of SiaN-VO with real flight data;
- Evaluate SiaN-VO on long routes and with more variations in flight dynamics;
- Measure the performance of SiaN-VO in the face of data failures and inconsistencies;
- Test our proposed model during a UAV flight, effectively embedding the network in the aircraft.

## Figures and Tables

**Table 1:** Details of the flights that make up the simulated dataset.

Environment	Map	Number of Routes
Downtown	A	50
Downtown	B	50
Downtown	C	50
Forest	A	12
Forest	B	10
Forest	C	50
Mountains	A	50
Mountains	B	50
Mountains	C	50

**Table 2:** Details of the name and size of each route used in the test stage.

Name	Flight Size (m)
Downtown1	346.03
Downtown2	674.80
Downtown3	458.49
Forest1	783.77
Forest2	321.01
Forest3	1001.24
Mountains1	906.62
Mountains2	784.24
Mountains3	569.84

**Table 3:** Summarization of results for flights with 20% predictions.

Route	Number of Predictions	Distance between Endpoints (m)	Average Distance between Expected and Predicted Position (m)
Downtown1	136	11.15	5.03
Downtown2	193	5.07	3.99
Downtown3	111	36.71	17.74
Forest1	72	1.17	6.28
Forest2	72	42.66	27.74
Forest3	83	19.14	21.17
Mountains1	197	13.32	21.17
Mountains2	275	52.84	43.68
Mountains3	195	55.71	48.33

**Table 4:** Summarization of results for flights with 40% predictions.

Route	Number of Predictions	Distance between Endpoints (m)	Average Distance between Expected and Predicted Position (m)
Downtown1	271	8.64	7.28
Downtown2	386	28.55	19.48
Downtown3	221	16.87	27.93
Forest1	143	1.00	5.94
Forest2	143	65.17	36.05
Forest3	166	36.05	23.79
Mountains1	393	22.46	23.33
Mountains2	550	94.10	56.95
Mountains3	390	58.78	34.87

**Table 5:** Summarization of results for flights with 60% predictions.

Route	Number of Predictions	Distance between Endpoints (m)	Average Distance between Expected and Predicted Position (m)
Downtown1	406	7.44	12.18
Downtown2	579	7.22	18.19
Downtown3	331	24.75	36.70
Forest1	214	15.96	16.82
Forest2	214	91.74	48.61
Forest3	249	34.53	19.67
Mountains1	589	28.07	32.06
Mountains2	825	129.05	69.73
Mountains3	585	49.05	49.85

**Table 6:** Summarization of results for flights with 100% predictions.

Route	Number of Predictions	Distance between Endpoints (m)	Average Distance between Expected and Predicted Position (m)
Downtown1	677	17.73	19.28
Downtown2	985	16.96	27.06
Downtown3	551	30.40	29.33
Forest1	357	15.58	12.23
Forest2	357	81.69	25.93
Forest3	415	48.94	28.81
Mountains1	981	22.82	15.31
Mountains2	1375	127.61	54.67
Mountains3	975	146.51	115.04

## Data Availability

The source code for this work can be accessed at https://github.com/verri/sian-vo (accessed on 1 January 2024), and the dataset for training, validation, and testing is available at http://doi.org/10.5281/zenodo.10140028.
